# Trabectedin and Radiation Therapy for Cardiac Metastasis From Leiomyosarcoma: A Case Report and Review of the Literature

**DOI:** 10.3389/fonc.2022.838114

**Published:** 2022-04-28

**Authors:** Ilaria Tortorelli, Federico Navarria, Antonio Di Maggio, Alberto Banzato, Chiara Lestuzzi, Luca Nicosia, Benedetta Chiusole, Antonella Galiano, Marta Sbaraglia, Vittorina Zagonel, Antonella Brunello

**Affiliations:** ^1^Oncology 1 Unit, Department of Oncology, Veneto Institute of Oncology IOV - IRCCS, Padua, Italy; ^2^Radiation Oncology Department, National Cancer Institute (CRO)- IRCCS, Aviano, Italy; ^3^Oncologic Radiology Unit, Department of Radiology and Medical Physics, Veneto Institute of Oncology IOV - IRCCS, Padua, Italy; ^4^Cardiology Unit, Veneto Institute of Oncology IOV - IRCCS, Padua, Italy; ^5^Cardiology and Cardio-Oncology Rehabilitation S.D.S, Department of Cardio-Cerebro-Vascular Physiopathology, Azienda Sanitaria Friuli Occidentale (AS FO), Aviano, Italy; ^6^Advanced Radiation Oncology Department, Sacro Cuore Don Calabria Hospital IRCCS, Negrar, Italy; ^7^Department of Pathology, Azienda Ospedale Università Padova, Padua, Italy

**Keywords:** leiomyosarcoma, radiotherapy, trabectedin, chemotherapy, cardiac metastasis

## Abstract

Leiomyosarcoma (LMS) is one of the most frequent subtypes of soft-tissue sarcomas (STSs). Metastatic spread to the heart in cancer patients carries a poor prognosis and there is no known effective treatment. Cardiac metastases of STSs are very rare. Here we present the case of a 55-year-old patient who underwent surgical resection of a retroperitoneal leiomyosarcoma and then developed widespread metastatic disease, treated with a combination of local treatment and systemic therapy. Three years after surgical resection she presented with a cardiac intraventricular mass, which was treated with radiation therapy, while receiving systemic therapy with trabectedin. Such combination therapy was well-tolerated and effective, allowing a substantial dimensional reduction which is perduring to date, 18 months after diagnosis of cardiac metastasis. Available literature and data point to the feasibility and good tolerability of radiation therapy and trabectedin in metastatic sarcoma, yet this is the first report on the effectiveness of the combination for the treatment of cardiac disease. The extended survival since a metastatic relapse (more than 3 years) is likely the result of integrated systemic and loco-regional treatment, which should be always discussed within the framework of a multiprofessional and multidisciplinary setting.

## Introduction

Leiomyosarcoma (LMS) is a malignant mesenchymal tumor that is derived from the smooth muscle lineage. It is one of the most frequent soft-tissue sarcoma (STS) subtypes, with an estimated incidence ranging between 10% and 20% of all newly diagnosed STSs (1). Compared to other sarcoma subtypes, LMS less commonly originates in the extremities, accounting for 10% to 15% of limb sarcomas; common primary sites include the retroperitoneum, abdomen, uterus, and the large blood vessels. In the retroperitoneal location, LMS is the second most common subtype, accounting for an estimated one third of cases and is the most common sarcoma arising from the large blood vessels in the retroperitoneum, namely the inferior vena cava and renal veins (1). Clinically, LMS presents with intrinsic biological aggressiveness and approximately 90% of LMS have a moderate to high grade. Indeed, patients with LMS have a decreased disease-specific survival compared to other histological subtypes and a higher risk for distant recurrence, with a high rate of pulmonary, liver, peritoneal, muscle, bone, and lymph node metastases (1).

Cardiac involvement in STSs is rare, with the most commonly involved subtypes being LMS, rhabdomyosarcoma, synovial sarcoma, alveolar soft-part sarcoma, clear cell sarcoma, and undifferentiated pleomorphic sarcoma (2). According to the limited available data LMS is the site of origin of cardiac metastasis in less than 5% of cases of cardiac involvement by malignant tumors. Autopsy reports have shown cardiac metastases to be present in as many as 25% of patients with STSs (3). The incidence of cardiac metastases has increased in the past decade because of longer survival rates of patients due to better diagnosis and for advanced STSs (4). Despite their frequency, cardiac metastases are often diagnosed during autopsy rather than in living patients, in light of their clinical presentation with non-specific symptoms and in some cases with asymptomatic clinical course. To date, only a few cases of cardiac metastases of sarcoma have been described and no uniform guidelines exist for their treatment.

We here discuss the case of a patient with metastatic LMS who presented with cardiac progression and was treated within a multidisciplinary approach which included concomitant systemic therapy with trabectedin and radiation therapy to the cardiac mass.

## Case Report

The patient is a 55-year-old woman, never-smoker, in good general health, with the only comorbidity being well-controlled hypertension treated with amlodipine.

**Figure 1:**
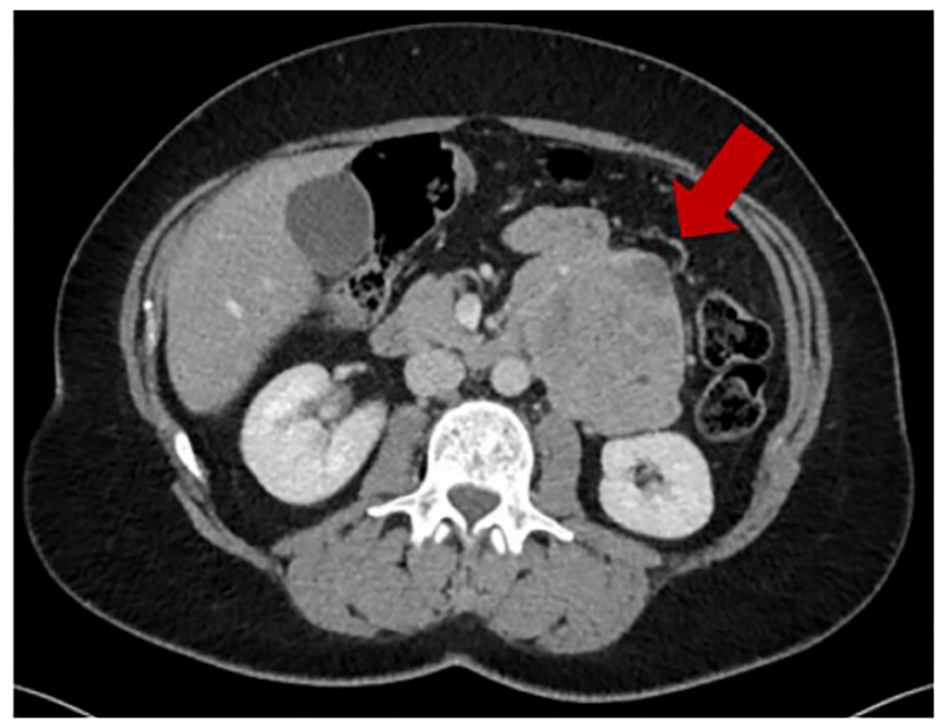
Baseline CT scan (16/December/2016).

In December 2016, because of onset of abdominal pain, an abdominal ultrasound was performed showing a mass in the retroperitoneal area. A chest and abdominal computed tomography (CT) scan revealed a large left retroperitoneal mass measuring 83 x 68 mm (**Figure 1**), with central necrosis. The mass was resected in February 2017, with histological diagnosis of LMS, grade 2 according to the French Federation of Cancer Centers Sarcoma Group (FNCLCC). Though the role of adjuvant chemotherapy in retroperitoneal leiomyosarcomas is debated, and controversies exist on the regimen to be used, the patient received adjuvant treatment with gemcitabine and dacarbazine from April 2017 to July 2017 at an outside facility.

She came to our attention with a restaging CT scan performed in November 2017 that showed a new 17x10 mm subcutaneous nodule in the right inferior abdominal wall, which was excised with pathologic confirmation of relapsed LMS.

In June 2018 two new subcutaneous nodules, respectively in the right lumbar region and on the lateral surface of the right leg, were detected during a restaging CT scan. A positron emission tomography (PET)-CT was also performed which revealed high glucose avidity of both nodules, which were then treated with stereotactic body radiation therapy (SBRT) at the total dose of 54 gray (Gy) and 48 Gy, respectively on lumbar and leg lesions, in 6 fractions.

At the next restaging CT scan in January 2019, a multifocal recurrence was detected with the appearance of multiple bilateral pulmonary nodules, the largest one in the left lower lobe measuring 6 mm, and of three new subcutaneous nodules, one in the median epigastrium, one in the left vulvar region and one in the suprapubic area, the largest one measuring 19 mm as the longest diameter. At that time, systemic treatment with doxorubicin and dacarbazine was started, with a partial response consisting of a dimensional reduction in all pulmonary nodules and in the epigastric and vulvar subcutaneous lesions, and stability in suprapubic nodule size. The patient’s primary and metastatic tumor (abdominal wall subcutaneous nodule) were tested with comprehensive genomic profiling, yet no actionable targets were found.

After completing 6 courses of chemotherapy, the patient underwent surgical resection of the subcutaneous nodules. The pathological report confirmed soft tissue localization of LMS. A new CT performed in December 2019 showed a dimensional increase in a single pulmonary nodule in the right middle lobe (9 mm Vs 2 mm), which was treated with SBRT in January 2020, with a total dose of 50 Gy in 5 fractions.

In February 2020 a restaging CT showed progressive disease, with an increased number and size of pulmonary nodules, the appearance of a right kidney nodule, and of a hypodense area in the left ventricle. A cardiac magnetic resonance imaging (MRI) was performed which confirmed the presence of a round, poorly-mobile mass in the left ventricular cavity measuring 20x17x15mm, with a necrotic core, between the anterolateral papillary muscle and anterolateral wall of the left ventricle. Also, a minor pericardial effusion was seen with overall left ventricular function maintained (ejection fraction: 59%).

**Figure 2:**
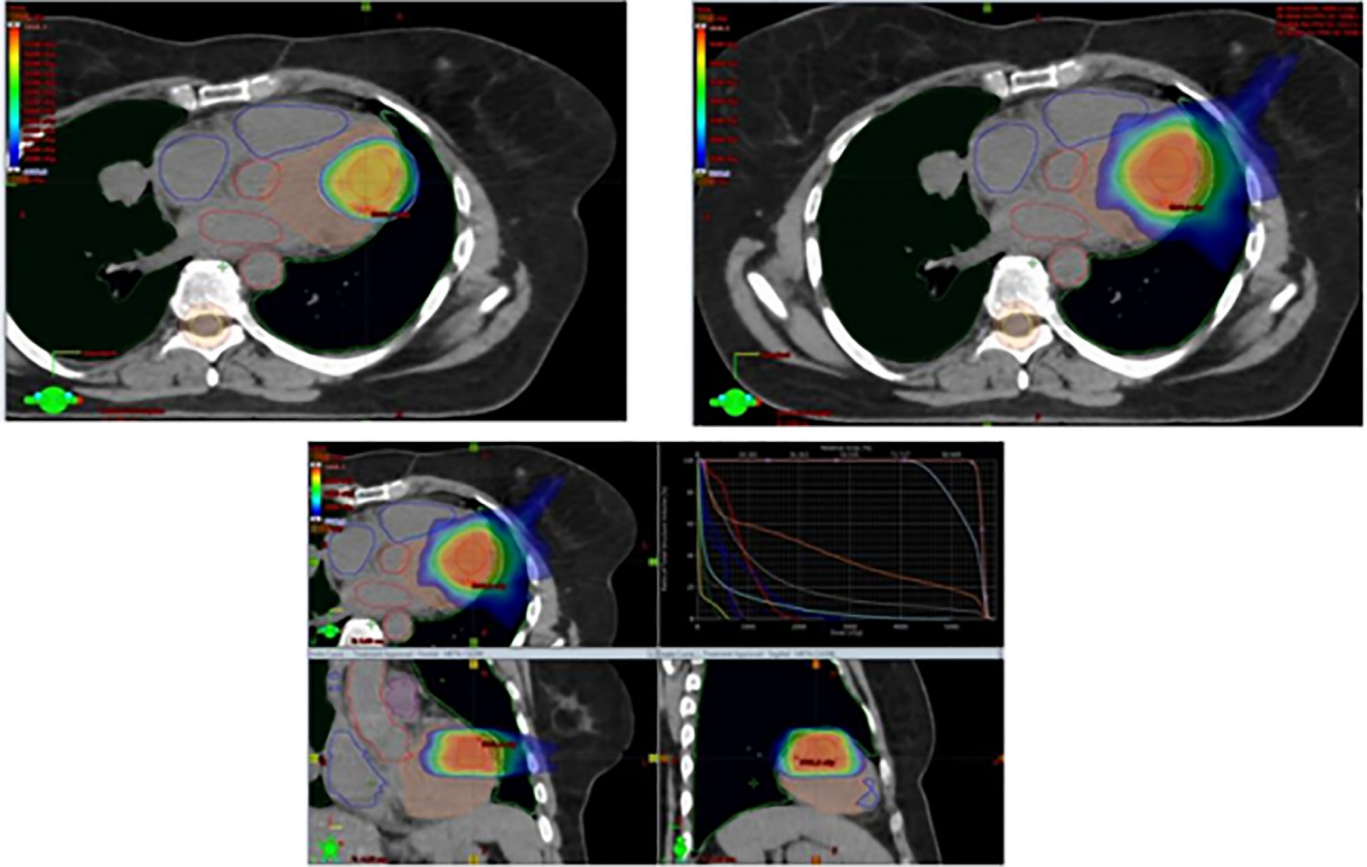
VMAT-IGRT for cardiac progression (CRO Aviano, Italy).

Being symptomatic for frequent episodes of arrhythmia, the patient was started on therapy with amiodarone. Due to multifocal progression, the patient was started on third-line systemic therapy with trabectedin 1.5 mg/mq as a 24-hour continuous infusion on day 1 of every 3-week cycles, and was evaluated for radiation therapy on the ventricular mass. Between May and June 2020, the patient received VMAT-IGRT (Volumetric Modulated Arc Therapy - Imaging Guided Radiation Therapy) to the ventricular lesion with 44Gy in 22 fractions to Planning Target Volume (PTV) plus simultaneous integrated boost to Gross Tumor Volume (GTV) at the dose of 55 Gy (**Figure 2**). Chemotherapy with trabectedin was maintained during the radiation treatment.

In the treatment plan for the management of organ motion and margin target-definition simulation, CT with respiratory gating, integrated with echocardiography, cardiac MRI, and PET-CT was used. During treatment, the patient was monitored with weekly clinical examinations, blood tests, electrocardiograms (ECG), Holter-ECGs, and echocardiography.

**Figure 3:**
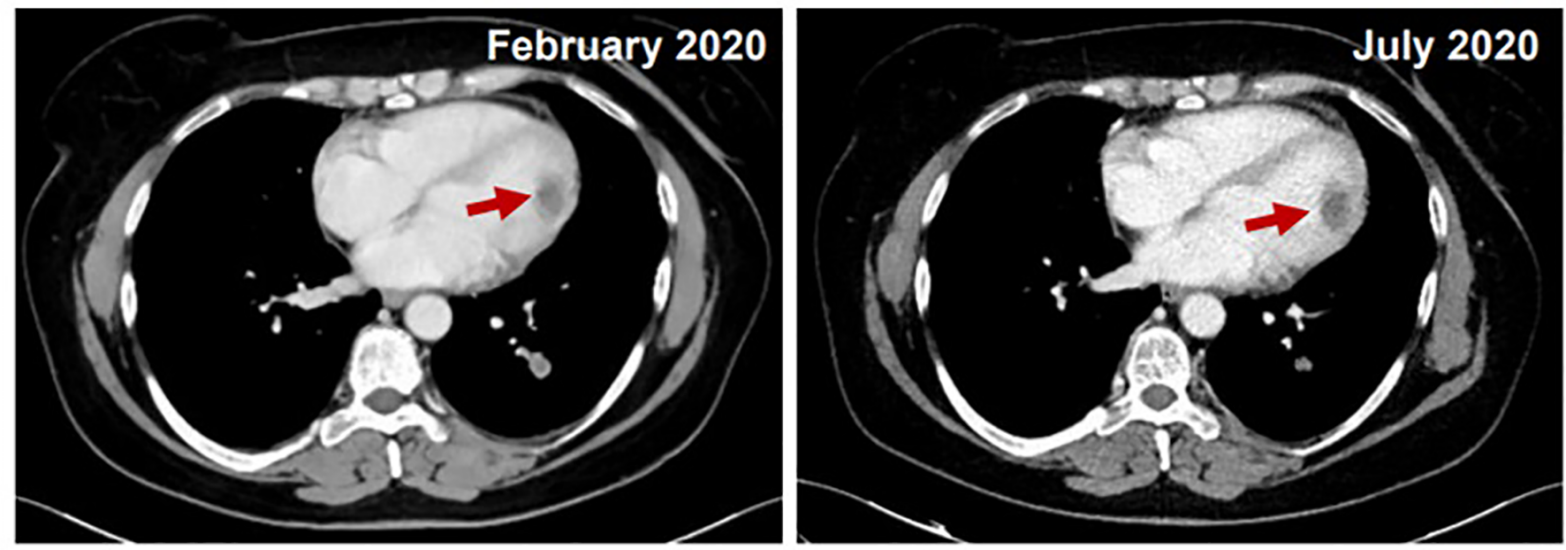
Comparison between February 2020 (on the left) and July 2020 (on the right): CT stability of cardiac mass and pulmonary and renal partial response.

At the end of radiation therapy no acute toxicity was seen. A restaging cardiac MRI performed after the end of radiotherapy showed a substantial stability of the mass in the left ventricular cavity, with an ejection fraction of 65%. A new restaging CT, performed in July 2020, showed pulmonary and renal partial response and no change of the cardiac mass (**Figure 3**). Since no further episodes of arrhythmia occurred, antiarrhythmic therapy was stopped. Trabectedin was continued at the same dose and schedule. Until September 2020 the patient received a total of 7 cycles of chemotherapy that were well tolerated except for grade 3 nausea, controlled with adjustment of anti-emetic prophylaxis; no hepatotoxicity was observed with dexamethasone pre- and post-medication. After 5 months from the start of trabectedin and radiation therapy, patient was in good general health with no cardiac-related symptoms.

**Figure 4:**
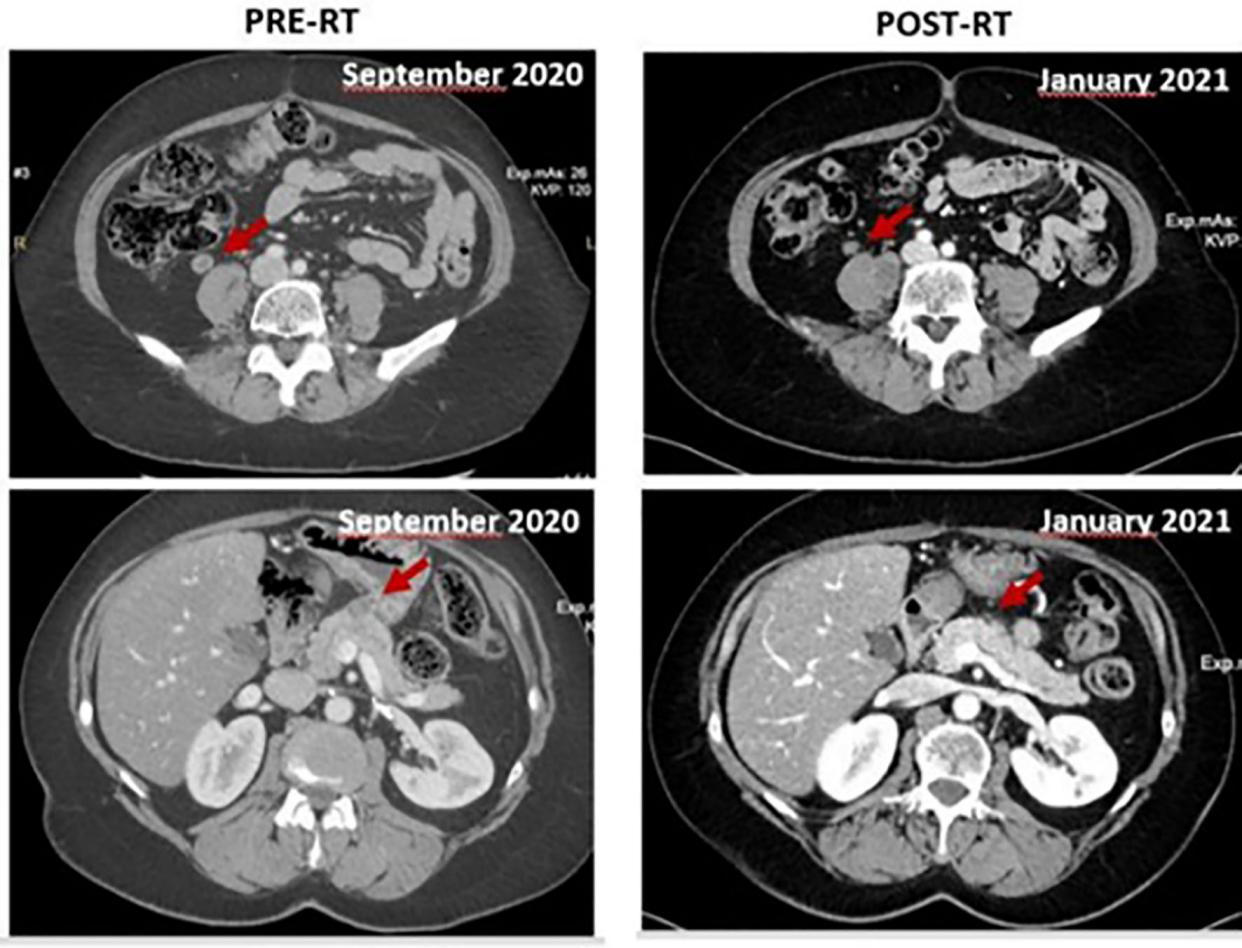
Comparison between September 2020 (on the left, pre-RT) and January 2021 (on the right, post-RT).

However, a new CT performed in September 2020, while showing partial response of the cardiac mass, revealed a slight dimensional increase of a pulmonary nodule in the right middle lobe (26 mm Vs 20 mm), of the nodule in the right kidney (17 mm Vs 14 mm) and a new pancreatic lesion of about 20 mm, confirmed by an abdominal MRI. Between October 2020 and November 2020, the patient received a new linac-based SBRT course to the lung lesion (56 Gy in 8 fractions) and 1.5 Tesla MR-guided SBRT on the pancreatic and renal lesions (45 Gy in 6 fractions and 45 Gy in 5 fractions, respectively) (5). In light of the multifocal progression, systemic therapy was switched to pazopanib. The CT performed in January 2021 showed stability of the cardiac mass (20 mm) and a partial response in the irradiated pulmonary (17 Vs 26 mm), pancreatic (14 Vs 20 mm) and renal (14 Vs 17 mm) lesions (**Figure 4**), and the patient continued treatment with pazopanib, with the disease stable at the next restaging CT scan in June 2021. A new echocardiogram, performed in July 2021, showed a fibrous residue instead of the previous tumor mass in the left ventricular cavity. At the restaging CT scan performed in September 2021, a multifocal disease progression was observed, with dimensional increase of pulmonary nodules in the left lower lobe (21 mm Vs 11 mm) and in the right lower lobe (14 mm Vs 10 mm) with the appearance of a left kidney nodule of about 30 mm. The left ventricular lesion was still responding and barely recognizable. A rechallenge with gemcitabine was attempted and radiation treatment on the renal and pulmonary lesions has been planned.

At the time of this writing, after more than 18 months since the diagnosis of cardiac metastasis, patient is in good general conditions with no new cardiac symptoms.

## Discussion

Patients with metastatic or unresectable STSs are generally considered incurable, except for some cases of isolated lung metastases, which can be approached with metastasectomy. In the advanced or metastatic stage, palliative systemic treatment is commonly used and the main goals of therapy are decrease of tumor bulk, reduction of symptoms, improvement of quality of life, and prolonging survival.

It is widely recognized that different histologic subtypes have variable patterns of chemosensitivity, with LMS displaying moderate sensitivity to chemotherapy, with different sensitivity to diverse agents (1, 6).

There is no established standard first-line chemotherapy, yet treatment to consider for first-line therapy include anthracycline-based regimens (doxorubicin alone, doxorubicin plus ifosfamide, doxorubicin plus dacarbazine). Gemcitabine-based regimens have been also used (i.e., gemcitabine plus docetaxel or gemcitabine plus dacarbazine), and could be an option in the case of contraindication to anthracyclines. Indeed, available evidence so far has shown doxorubicin alone to be as effective as the combination of gemcitabine and docetaxel, and less toxic (7). Second and further-lines of treatment are commonly delivered with the aim of pursuing long-term disease control at the lowest possible toxicity level. They include several agents such as ifosfamide, gemcitabine, dacarbazine, and taxanes used on the basis of sarcoma histological subtype, though the optimal treatment sequence for the management of metastatic disease has not yet been defined (8, 9). Within the group of targeted therapies, the multitargeted tyrosine kinase inhibitor pazopanib has shown some activity in previously treated non-adipocytic STSs, improving progression-free survival (PFS) compared to placebo (10). Other drugs such as trabectedin and eribulin have been approved for patients with adipocytic sarcomas, showing greater effectiveness than dacarbazine in randomized trials on advanced STSs (11, 12). Trabectedin represents an innovative treatment option, with several mechanisms of action, including antiangiogenic and cytotoxic effects. It has been approved as a treatment option for patients with STSs in progression after first-line chemotherapy with anthracyclines or for those unfit to receive these agents and it has shown positive results in patients with lipo- and leiomyosarcomas (the so-called “L-sarcomas”) (1, 12). In addition, some retrospective and randomized trials have shown the antitumor activity of trabectedin in non-L-type sarcomas, such as synovial sarcoma and solitary fibrous tumors (SFTs) (13–16). Moreover, some clinical trials studying the combination of trabectedin and other agents are ongoing. Among them, a multicenter randomized phase II trial is studying the efficacy and safety of trabectedin and the Poly (ADP-ribose) polymerase (PARP)-inhibitor olaparib compared with trabectedin in monotherapy in patients with advanced, metastatic, or unresectable STSs after failure of standard treatments (NCT03838744). Another ongoing multicenter phase I trial is evaluating the efficacy and safety of trabectedin and metronomic cyclophosphamide in management of advanced pretreated STSs (NCT02805725). The combination of doxorubicin with trabectedin seems particularly active in LMSs, and following encouraging results of a phase II trial (17), a randomized phase III study comparing the combination of doxorubicin plus trabectedin with doxorubicin alone in first-line therapy in metastatic LMSs (NCT02997358) has been conducted. Preliminary data have been presented at the 2021 ESMO meeting which showed a significant improvement in PFS of the combination vs Doxorubicin single agent (13.5 vs 7.3 months, with adjusted HR 0.384, p<.0001), with a non-statistically significant improvement of overall survival as well, at the cost of additional expected yet manageable toxicity (18).

In recent years, evidence of the role of loco-regional treatments in the approach to advanced stage disease has been accumulating (19, 20). This is mainly based on a number of retrospective studies, often with limited sample sizes, and involves primarily pulmonary metastasectomy (21–25).

A large retrospective study by the French Sarcoma Group evaluated prognostic factors, outcomes, and patterns of care of a cohort of 2,225 patients with metastatic STS diagnosed between 1990 and 2013. Out of 2,225 patients, 1,054 (49%) patients underwent locoregional treatment of metastases. Patients with LMS had a better outcome compared to patients with other histological subtypes. With the exception of LMSs, there was a very limited benefit of lines of therapies beyond the third-line, with a median time to next treatment and a median overall survival ranging between 5.4 and 8.5 months, respectively. Importantly, more than 80% of patients in this series who were alive 5 years after the diagnosis of metastatic disease had received a loco-regional treatment, suggesting that radiofrequency ablation, surgery, and a combination of different approaches are beneficial in terms of survival (26).

However, to date, the best integration of locoregional treatment of metastases in the management of advanced STSs has not yet been defined.

Management of such patients, provided by a multi-professional specialized team, can lead to integrated treatment that can prove very effective, even in highly complex clinical situations such as cardiac metastases.

## Cardiac Metastases From Sarcoma

The spread of cardiac metastases is a life-threatening condition due to cardiac failure within short time from its diagnosis. Although primary cardiac tumors are uncommon (post mortem studies report a frequency between 0.001% and 0.28%), secondary tumors are not (4, 27) and the incidence of cardiac metastases reported in literature ranges from 2.3% and 18.3% (27–29). Primary malignancies associated with cardiac metastases are mainly carcinomas, but also lymphomas, leukemias, and melanomas can spread to the heart (27).

Metastatic heart disease is indeed a difficult condition to diagnose since its clinical features are not well defined. The most common symptoms of neoplastic involvement of the heart may be new valve disease, heart failure, arrhythmias, conduction defects, syncope or pulmonary embolus, and even sudden death, however at first they are often asymptomatic (30). To date, there are no uniform guidelines for the treatment of cardiac metastases. According to the limited available data, surgery, when feasible, should be performed to prevent life-threatening conditions and cardiac transplantation may represent an emerging strategy in cases of isolated unresectable cardiac involvement. Chemotherapy and/or radiotherapy have been demonstrated to prolong survival in a few patients with unresectable cardiac lesions (31, 32).

Cardiac metastases from sarcoma are uncommon and only a few cases have been described to date. In 1986, Hallahan et al. reviewed autopsy reports of 120 patients with metastatic STS from 1940 to 1986. Cardiac metastases were present in 25% of consecutive autopsies; 33% of patients had metastases to the pericardium, while 50% had myocardial metastases, and 17% had both. Ten patients had congestive heart failure and other signs and symptoms of cardiac metastases from STS included: conduction block (two), arrhythmias (two), chest pain (three), simulation of an atrial myxoma (one), and sudden death (one). However, the majority of metastases in those reports were not diagnosed before autopsy, leading to the hypothesis that cardiac metastases from STS are generally uncommon and asymptomatic (3).

In 2010, Takenaka et al. reviewed the data of 641 patients with STS treated at Osaka Medical Center for Cancer and Cardiovascular Disease between 1996 and 2009, among which 11 patients had cardiac metastases diagnosed while they were alive. The median age at diagnosis was 33 years. The median interval from diagnosis to cardiac metastasis was 36 months (8-108 months). The most common primary tumor was leiomyosarcoma (n=5), followed by rhabdomyosarcoma, alveolar soft-part sarcoma, clear cell sarcoma, undifferentiated pleomorphic sarcoma and synovial sarcoma. Metastases to myocardium were more common in the left side of the heart, in particular in the left ventricle. In all cases, metastases were found in other organs before or concurrently with the cardiac involvement. Echocardiography led to the definitive diagnosis in six cases, with four cases diagnosed by CT. In one case, pericardial metastasis was found accidentally during resection of lung metastasis. Symptoms at diagnosis of cardiac involvement included dyspnea (five cases), and fatigue (two cases), with four patients asymptomatic. In three patients a continuous drainage of pericardial fluid was required and two of these three patients had an increase of pericardial effusion during chemotherapy.

To the best of our knowledge, no data are available with regard to possible molecular markers associated with sarcoma metastases to the heart. Radiotherapy was used to treat seven cases of cardiac metastasis, while surgery was not used in any patient. Survival from diagnosis of cardiac metastasis was 8 months on average. Patients treated with radiation therapy had a median survival of 10.5 months, compared with 3.5 months for patients who did not receive radiation therapy (2).

In 2012, Agaimy et al. evaluated retrospectively patients undergoing cardiac surgery for primary or secondary cardiac sarcoma in the period 1999-2011 at the Erlangen Heart Centre in Germany. Four patients had cardiac metastases from STS (namely myxoid liposarcoma, pleomorphic spindle cell sarcoma, osteosarcoma, and alveolar soft part sarcoma). The median interval from initial diagnosis to cardiac metastases from STS was 109.5 months (range 5-240 months). Three patients died of disease at a mean of 14 months after cardiac surgery and one was disease free 34 months after heart transplantation for metastasis (31).

In **Table 1** we report the cases described in literature, with treatment performed and outcomes, where available.

**Table 1:** Cardiac metastases from sarcoma.

Author	N	Age	Sex (M/F)	Histological Subtypes (Leiomyosarcoma/Other)		Metachronous/Synchronous		Site (pericardial/m yocardial/endo cardial)			Symptoms	DFI (months)	Surgery	RT		CT/Other	Regimen	Survival from heart met (months)	PFS (months)	OS (months)	
**Orsmond et al. (1976)** (32)	1	12	F	Rhabdomyosarcoma		Metachronous		Myocardial (IS, LV)			Syncope and angina	72	Metastasis resection	35 Gy			Actinomycin D, Vincristine, oral cyclophosphamide	N/A (alive and well 9 months after surgery)	N/A	N/A	
**Hammond et al. (1976)** (33)	1	69	F	Occult Chondrosarcoma (found at autopsy)		N/A		Myocardial (LA)			Dyspnea and palpitations	N/A	Metastasis resection				N/A	26	14	26	
**Dash et al. (1983)** (34)	1	25	F	Osteosarcoma		Metachronous		Myocardial (LA)			Dyspnea	60	Metastasis resection				N/A	1	1	61	
**Martin et al. (1983)** (35)	1	30	F	Leiomyosarcoma		Metachronous		Myocardial (LV)			Fatigue and dyspnea	11	Metastasis resection				N/A	0	0	11	
**Tominaga et al. (1986)** (36).	1	25	M	Chordoma		Metachronous		Myocardial (RV)			Dyspnea	108				Chemotherapy	N/A	48	48	156	
**Hallahan et al. (1986)** (3)	1	54	F	Leiomyosarcoma		Metachronous		Myocardial (LA)			Dyspnea, chest tightness in recurrent transient	36	Metastasis resection				N/A	9	6	42	
									Ischemic attacks												
	2	77	F	Undifferentiated pleomorphic sarcoma		Synchronous		Myocardial (RV)	Asymptotic		0						N/A	0	0	0	
**Chalmers et al. (1987)** (37)	1	40	F	Endometrial stromal sarcoma		Metachronous		Endocardial (LA; found at autopsy)	Frequent hemiparesis and aphasia in recurrent and ultimately fatal cerebral embolic strokes		22		N/A	N/A	N/A		N/A	N/A	N/A	N/A	
**Limper et al. (1988)** (38)	1	32	M	Osteosarcoma		Metachronous		Myocardial (LA)	Left hemiplegia in acute cerebral infarction		96		Metastasis resection		Chemotherapy		Bleomycin, Cyclophosphamide, Dactinomycin, and Methotrexate with Leucovorin	4	4	100	
**Khoo et al. (1997)** (39)	1	57	M	Synovial Sarcoma		Metachronous		Myocardial (LV)	Right hand paresthesia, pain and coldness in acute ischemic arm		56						N/A	4	4	60	
**Locci et al. (2001)** (40)	1	29	M	Gastric sarcoma		Metachronous		Myocardial (LA)	Dyspnea		1		Metastasis resection		Chemotherapy		N/A	11	1	12	
**Cuadrado et al. (2007)** (41)	1	74	F	Solitary fibrous tumor	Metachronous	Myocardial (LA)	Dyspnea	72	Metastasis resection					N/A	6		6			78	
**Takenaka et al. (2011)** (2)	1	40	F	Leiomyosarcoma	Metachronous	Myocardial (LA)	Dyspnea	65						N/A	0		0			65	
	2	55	F	Leiomyosarcoma Metachronous Myocardial Fatigue (RV)				36	**-**	25G	DCE			N/A	24		N/A			N/A	
										y/5fr											
	3	63	F	Leiomyosarcoma	Metachronous	Myocardial (LV)	Asymptom	108		45 G				N/A	13		N/A			N/A	
										y/15f r											
	4	47	M	Leiomyosarcoma	Metachronous	Myocardial (LV)	Dyspnea	30		50G				N/A	7		N/A			N/A	
										y/25f r											
	5	26	F	Leiomyosarcoma	Metachronous	Myocardial (LV)	Asymptom	51		60G				N/A	18		N/A			N/A	
										y/30f r											
	6	30	F	Clear cell sarcoma	Metachronous	Myocardial (LV)	Asymptom	31	**-**	**-**	Immunothe rapy			N/A	4		N/A			N/A	
	7	**33**	F	Clear cell sarcoma	Metachronous	Pericardium	Asymptom	64	**-**	**-**	**-**			N/A	5		N/A			N/A	
	8	23	M	Alveolar soft part sarcoma	Metachronous	Myocardial (LV)	Asymptom	27		60G	Chemother apy				8		N/A			N/A	
										y/30f r											
	9	69	F	Undifferentiated pleomorphic sarcoma	Metachronous	Myocardial (RA)	Dyspnea	13	**-**	**-**	**-**			N/A	5		N/A			N/A	
	1 0	16	M	Rhabdomyosarcoma	Metachronous	Pericardium	Fatigue	8		40G	Chemother apy			N/A	6		N/A			N/A	
										y/20f r											
	1 1	13	F	Synovial sarcoma	Metachronous	Pericardium	Dyspnea	57		32G	Chemother apy/DCE/P ericardioto my			N/A	5		N/A			N/A	
										y/16fr											
**Agaimy et al. (2012)** (31)	1	36	F	Osteosarcoma	Metachronous	Myocardial (LA)	Functional Mitral stenosis	156	Metastasis resection					N/A	10		10			166	
	2	31	F	Alveolar soft part sarcoma	Metachronous	Myocardial (IS,LV)	Paroxysmal cardiac pain	240	Cardiac Transplanta tion					N/A	N/A (alive and well 34 months after HTX)		N/A			N/A	
	3	38	F	Spindle cell sarcoma	Metachronous	Myocardial (LA, MV)	Asymptom	5	Metastasis resection	_	_			N/A	30		30			35	
	4	62	M	Myxoid liposarcoma	Metachronous Myocardial Asymptom 36 (RA) + IVC				Metastasis resection	_	_			N/A	2		2			38	
**Park et al. (2016)** (42)	1	54	M	Leyomiosarcoma	Metachronous	Myocardial (IS)	Dizziness and dyspnea in AV block	60			Chemother apy/Perman ent pacemaker			Pazopanib	3		3			63	
**Martinez et al. (2017)** (43)	1	63	M	Leiomyosarcoma	Metachronous	Myocardial (RV)	Dyspnea	14		Unsp ecifi ed dose	DCE			N/A	5		5			19	
Tortorelli et al. (2022)	1	54	F	Leiomyosarcoma	Metachronous	Myocardial (LV)	Frequent episodes of arrhythmia	36		Tom Other apy (44Gy/22fr)	Chemother apy			Trabectedin	N/A (alive and well 11 months after heart met)		N/A			N/A	

DCE, drainage of cardiac effusion; LA, left atrium; N/A, not available; RA, Right atrium; RV, Right ventricle; IS, interventricular septum.

A key message is that the diagnosis of cardiac metastases should always be considered for patients with STSs with sudden onset of cardiac dysfunction. Palliative radiotherapy or chemotherapy may confer a symptomatic benefit and a better outcome in terms of overall survival. In selected patients with isolated cardiac metastasis, surgery could be considered. Moreover, since patients with metastatic STS undergo several restaging CT scans during the course of disease, the heart should always be thoroughly assessed for the presence of asymptomatic localization of sarcoma (2, 3, 31–43).

To the best of our knowledge, the present report describes the first case of cardiac intracavitary metastasis of STS treated with trabectedin in combination with radiation therapy to the cardiac mass, with significant efficacy and minimal toxicity.

## Trabectedin and Concomitant Radiation

In our patient, we ultimately used concomitant chemotherapy with trabectedin, along with radiation therapy given to the heart lesion. The rationale for the association of systemic treatment with trabectedin and radiotherapy in an unresectable cardiac metastasis is derived from preclinical data demonstrating a radio-sensitizing effect of trabectedin (44).

Trabectedin is a marine-derived antineoplastic agent, first approved in Europe in 2007, which has shown particular efficacy in myxoid liposarcoma, and in general, in liposarcoma and leiomyosarcoma. Its mechanism of action resides in binding to specific triplets of the DNA minor groove, affecting the activity of DNA binding proteins, including some DNA repair pathways, with an alteration of the cell cycle and eventual cell death. In addition to direct effect against tumor cells, it also has host-modulating effects of great importance for its therapeutic activity. As revealed by preclinical and clinical evidence, this drug is able to decrease the number of tumor-associated macrophages (TAMs) and to modify the tumor angiogenesis and microenvironment at therapeutic doses (12, 44–48).

Trabectedin, at pharmacologically appropriated concentrations, has shown to have an important *in vitro* radio-sensitizing effect, inducing cell cycle alterations and death in several human tumor cell lines. According to cell kinetic studies, trabectedin would sensitize sarcoma cells to radiation by synchronizing cells in the G2/M phase, the most sensitive to radiation (44).

Based on these preclinical data, a phase I/II clinical trial (TRASTS study) evaluated the synergistic effect of trabectedin plus radiotherapy in patients with progressing metastatic, non-resectable STS after at least one previous line of systemic therapy. In this study, patients with lung metastases from STS were treated with trabectedin at the standard dose of 1.5 mg/m2 every three weeks in a 24-hour infusion in combination with radiotherapy on pulmonary nodules at a fixed dose of 30 Gy in 10 daily fractions, started within one hour at the end of the trabectedin infusion. Trial results showed an overall response rate of 60%, with a median 6-months PFS rate of 75% and a median 6-month OS of 86%. Interestingly, the combination had a favorable safety profile, except for three cases of acute radiation pneumonitis (49, 50).

In our patient, we were able to deliver 44 Gy in 22 fractions, a 55 Gy simultaneous integrated boost to the GTV with a VMAT-IGRT technique, and a standard schedule of trabectedin with no relevant toxicity plus significant efficacy. Indeed, a VMAT-IGRT technique until the dose of 55 Gy on the cardiac metastasis was preferred over a prolonged SBRT with 30 Gy in 10 fractions in light of the excellent performance status of the patient and the oligometastatic progression, associated with chemotherapy in order to provide systemic control of the disease.

## Conclusion

Cardiac metastases from STSs represent a rare, life-threatening condition, with a great heterogeneity in clinical presentation and course of disease. They are often insidious and must always be included in the differential diagnosis of patients presenting with new cardiac symptoms, though cardiac metastases are often asymptomatic. Restaging imaging should be always thoroughly assessed also at cardiac site, and echocardiography as well as more specific imaging techniques such as cardiac MRI may be of help in diagnosing cardiac metastases.

Even if to date no uniform guidelines exist for their treatment, surgery whenever feasible should be recommended to prevent life-threatening complications. Chemotherapy and/or radiation therapy can be used with the aim of survival improvement. A concomitant chemo-radiation approach is feasible, and since most metastatic cardiac disease in STS is from LMS, trabectedin appears a very good option for systemic treatment in combination with radiation therapy, leading to a potent synergistic effects with long-lasting results, in our case allowing for complete clinical and radiological response to the cardiac lesion.

## Data Availability Statement

The original contributions presented in the study are included in the article. Further inquiries can be directed to the corresponding author.

## Ethics Statement

The studies involving human participants were reviewed and approved by the Ethics Committee Istituto Oncologico Veneto. The patients/participants provided their written informed consent to participate in this study.

## Author Contributions

AB, FN, IT, conceptualization, methodology, formal analysis, supervision, and validation. FN, IT, LN, ABa, AB data curation, investigation, validation. CL, BC, AG, VZ, formal analysis, investigation, validation. MS, FN, IT data curation, investigation. AB, VZ, supervision and validation. AB, IT, methodology, supervision, validation. All authors contributed to the article and approved the submitted version.

## Conflict of Interest

ABr declares consulting fees from GlaxoSmithKline; PharmaMar; Eisai; Roche; travel grants from Takeda; Pharmamar.

The remaining authors declare that the research was conducted in the absence of any commercial or financial relationships that could be construed as a potential conflict of interest.

## Publisher’s Note

All claims expressed in this article are solely those of the authors and do not necessarily represent those of their affiliated organizations, or those of the publisher, the editors and the reviewers. Any product that may be evaluated in this article, or claim that may be made by its manufacturer, is not guaranteed or endorsed by the publisher.

## References

[1] Serrano C George S. Leiomyosarcoma. Hematol Oncol Clin North Am (2013) 27(5):957–74. doi: 10.1016/j.hoc.2013.07.002 24093170

[2] Takenaka S Hashimoto N Araki N Hamada K Naka N Joyama S. Eleven Cases of Cardiac Metastases From Soft-Tissue Sarcomas. Jpn J Clin Oncol (2011) 41(4):514–8. doi: 10.1093/jjco/hyq246 21247968

[3] Hallahan DE Vogelzang NJ Borow KM Bostwick DG Simon MA. Cardiac Metastases From Soft-Tissue Sarcomas. J Clin Oncol (1986) 4(11):1662–9. doi: 10.1200/JCO.1986.4.11.1662 3772419

[4] Bussani R De-Giorgio F Abbate A Silvestri F. Cardiac Metastases. J Clin Pathol (2007) 60(1):27–34. doi: 10.1136/jcp.2005.035105 17098886 PMC1860601

[5] Nicosia L Sicignano G Rigo M Figlia V Cuccia F De Simone A. Daily Dosimetric Variation Between Image-Guided Volumetric Modulated Arc Radiotherapy and MR-Guided Daily Adaptive Radiotherapy for Prostate Cancer Stereotactic Body Radiotherapy. Acta Oncol (2021) 60(2):215–21. doi: 10.1080/0284186X.2020.1821090 32945701

[6] Bathan AJ Constantinidou A Pollack SM Jones RL. Diagnosis, Prognosis, and Management of Leiomyosarcoma: Recognition of Anatomic Variants. Curr Opin Oncol (2013) 25(4):384–9. doi: 10.1097/CCO.0b013e3283622c77 23635801

[7] Seddon B Strauss SJ Whelan J Leahy M Woll PJ Cowie F. Gemcitabine and Docetaxel Versus Doxorubicin as First-Line Treatment in Previously Untreated Advanced Unresectable or Metastatic Soft-Tissue Sarcomas (GeDDiS): A Randomised Controlled Phase 3 Trial. Lancet Oncol (2017) 18(10):1397–410. doi: 10.1016/S1470-2045(17)30622-8 PMC5622179 28882536

[8] García-Del-Muro X López-Pousa A Maurel J Martín J Martínez-Trufero J Casado A. Randomized Phase II Study Comparing Gemcitabine Plus Dacarbazine Versus Dacarbazine Alone in Patients With Previously Treated Soft Tissue Sarcoma: A Spanish Group for Research on Sarcomas Study. J Clin Oncol (2011) 29(18):2528–33. doi: 10.1200/JCO.2010.33.6107 21606430

[9] Le Cesne A Antoine E Spielmann M Le Chevalier T Brain E Toussaint C. High-Dose Ifosfamide: Circumvention of Resistance to Standard-Dose Ifosfamide in Advanced Soft Tissue Sarcomas. J Clin Oncol (1995) 13(7):1600–8. doi: 10.1200/JCO.1995.13.7.1600 7541449

[10] Van der Graaf WT Blay JY Chawla SP Kim DW Bui-Nguyen B Casali PG. Pazopanib for Metastatic Soft-Tissue Sarcoma (PALETTE): A Randomised, Double-Blind, Placebo-Controlled Phase 3 Trial. Lancet (2012) 379(9829):1879–86. doi: 10.1016/S0140-6736(12)60651-5 22595799

[11] Schöffski P Chawla S Maki RG Italiano A Gelderblom H Choy E. Eribulin Versus Dacarbazine in Previously Treated Patients with Advanced Liposarcoma or Leiomyosarcoma: A Andomised, Open-Label, Multicentre, Phase 3 Trial. Lancet (2016) 387(10028):1629–37. 10.1016/S0140-6736(15)01283-0 26874885

[12] Demetri GD von Mehren M Jones RL Hensley ML Schuetze SM Staddon A. Efficacy and Safety of Trabectedin or Dacarbazine for Metastatic Liposarcoma or Leiomyosarcoma After Failure of Conventional Chemotherapy: Results of a Phase III Randomized Multicenter Clinical Trial. J Clin Oncol (2016) 34(8):786–93. doi: 10.1200/JCO.2015.62.4734 PMC5070559 26371143

[13] Khalifa J Ouali M Chaltiel L Le Guellec S Le Cesne A Blay JY. Efficacy of Trabectedin in Malignant Solitary Fibrous Tumors: A Retrospective Analysis From the French Sarcoma Group. BMC Cancer (2015) 15:700. doi: 10.1186/s12885-015-1697-8 26472661 PMC4608145

[14] Le Cesne A Cresta S Maki RG Blay JY Verweij J Poveda A. A Retrospective Analysis of Antitumour Activity With Trabectedin in Translocation-Related Sarcomas. Eur J Cancer (2012) 48(16):3036–44. doi: 10.1016/j.ejca.2012.05.012 22749255

[15] Sanfilippo R Dileo P Blay JY Constantinidou A Le Cesne A Benson C. Trabectedin in Advanced Synovial Sarcomas: A Multicenter Retrospective Study From Four European Institutions and the Italian Rare Cancer Network. Anticancer Drugs (2015) 26(6):678–81. doi: 10.1097/CAD.0000000000000228 PMC4730787 25763543

[16] De Sanctis R Marrari A Marchetti S Mussi C Balzarini L Lutman FR. Efficacy of Trabectedin in Advanced Soft Tissue Sarcoma: Beyond Lipo- and Leiomyosarcoma. Drug Des Devel Ther (2015) 9:5785–91. doi: 10.2147/DDDT.S92395 PMC4629957 26604682

[17] Pautier P Floquet A Chevreau C Penel N Guillemet C Delcambre C. Trabectedin in Combination With Doxorubicin for First-Line Treatment of Advanced Uterine or Soft-Tissue Leiomyosarcoma (LMS-02): A Non-Randomised, Multicentre, Phase 2 Trial. Lancet Oncol (2015) 16(4):457–64. doi: 10.1016/S1470-2045(15)70070-7 25795402

[18] Pautier P Italiano A Piperno-Neumann S Chevreau CM Penel N Cupissol D. LMS-04 Study: A Randomised, Multicenter, Phase III Study Comparing Doxorubicin Alone Versus Doxorubicin With Trabectedin Followed by Trabectedin in Non-Progressive Patients as First-Line Therapy, in Patients With Metastatic or Unresectable Leiomyosarcoma - A French Sarcoma Group study. Ann Oncol (2021) 32 suppl_5):S1283–346. doi: 10.1016/annonc/annonc741

[19] O'Sullivan B Davis AM Turcotte R Bell R Catton C Chabot P. Preoperative Versus Postoperative Radiotherapy in Soft-Tissue Sarcoma of the Limbs: A Randomised Trial. Lancet (2002) 359(9325):2235–41. doi: 10.1016/S0140-6736(02)09292-9 12103287

[20] Saponara M Stacchiotti S Casali PG Gronchi A. (Neo)adjuvant Treatment in Localised Soft Tissue Sarcoma: The Unsolved Affair. Eur J Cancer (2017) 70:1–11. doi: 10.1016/j.ejca.2016.09.030 27866094

[21] Okiror L Peleki A Moffat D Bille A Bishay E Rajesh P. Survival Following Pulmonary Metastasectomy for Sarcoma. Thorac Cardiovasc Surg (2016) 64(2):146–9. doi: 10.1055/s-0035-1546430 25742552

[22] Marulli G Mammana M Comacchio G Rea F. Survival and Prognostic Factors Following Pulmonary Metastasectomy for Sarcoma. J Thorac Dis (2017) 9(Suppl 12):S1305–15. doi: 10.21037/jtd.2017.03.177 PMC5653498 29119019

[23] Smith R Pak Y Kraybill W Kane JM 3rd. Factors Associated With Actual Long-Term Survival Following Soft Tissue Sarcoma Pulmonary Metastasectomy. Eur J Surg Oncol (2009) 35(4):356–61. doi: 10.1016/j.ejso.2008.01.004 18294807

[24] Dudek W Schreiner W Mykoliuk I Higaze M Sirbu H. Pulmonary Metastasectomy for Sarcoma-Survival and Prognostic Analysis. J Thorac Dis (2019) 11(8):3369–76. doi: 10.21037/jtd.2019.08.10 PMC6753416 31559040

[25] Yamamoto Y Kanzaki R Kanou T Ose N Funaki S Shintani Y. Long-Term Outcomes and Prognostic Factors of Pulmonary Metastasectomy for Osteosarcoma and Soft Tissue Sarcoma. Int J Clin Oncol (2019) 24(7):863–70. doi: 10.1007/s10147-019-01422-0 30806840

[26] Savina M Le Cesne A Blay JY Ray-Coquard I Mir O Toulmonde M. Patterns of Care and Outcomes of Patients With METAstatic Soft Tissue SARComa in a Real-Life Setting: The METASARC Observational Study. BMC Med (2017) 15(1):78. doi: 10.1186/s12916-017-0831-7 28391775 PMC5385590

[27] Mukai K Shinkai T Tominaga K Shimosato Y. The Incidence of Secondary Tumors of the Heart and Pericardium: A 10-Year Study. Jpn J Clin Oncol (1988) 18(3):195–201. doi: 10.1093/oxfordjournals.jjco.a039238 3411785

[28] Díaz ML Villanueva A Bastarrika G Zudaire B del Barrio LG Noguera JJ. Non-Electrocardiogram-Gated Multidetector-Row Computed Tomography Findings of Cardiac Pathology in Oncologic Patients. Curr Probl Diagn Radiol (2009) 38(5):206–17. doi: 10.1067/j.cpradiol.2008.05.003 19632498

[29] Strecker T Rösch J Weyand M Agaimy A. Primary and Metastatic Cardiac Tumors: Imaging Characteristics, Surgical Treatment, and Histopathological Spectrum: A 10-Year-Experience at a German Heart Center. Cardiovasc Pathol (2012) 21(5):436–43. doi: 10.1016/j.carpath.2011.12.004 22300501

[30] Freedberg RS Kronzon I Rumancik WM Liebeskind D. The Contribution of Magnetic Resonance Imaging to the Evaluation of Intracardiac Tumors Diagnosed by Echocardiography. Circulation (1988) 77(1):96–103. doi: 10.1161/01.cir.77.1.96 3335075

[31] Agaimy A Rösch J Weyand M Strecker T. Primary and Metastatic Cardiac Sarcomas: A 12-Year Experience at a German Heart Center. Int J Clin Exp Pathol (2012) 5(9):928–38. PMC3484490 23119110

[32] Orsmond GS Knight L Dehner LP Nicoloff DM Nesbitt M Bessinger FB Jr. Alveolar Rhabdomyosarcoma Involving the Heart. An Echocardiographic, Angiographic and Pathologic Study. Circulation (1976) 54(5):837–43. doi: 10.1161/01.cir.54.5.837 975481

[33] Hammond GL Strong WW Cohen LS Silverman M Garnet R LiVolsi VA. Chondrosarcoma Simulating Malignant Atrial Myxoma. J Thorac Cardiovasc Surg (1976) 72(4):575–80. doi: 10.1016/S0022-5223(19)40043-3 966789

[34] Dash H Little JR Zaino R Colao DJ Chaurushiya P Schoolwerth AC. Metastatic Periosteal Osteosarcoma Causing Cardiac and Renal Failure. Am J Med (1983) 75(1):145–9. doi: 10.1016/0002-9343(83)91178-6 6574700

[35] Martin JL Boak JG. Cardiac Metastasis From Uterine Leiomyosarcoma. J Am Coll Cardiol (1983) 2(2):383–6. doi: 10.1016/s0735-1097(83)80180-6 6863772

[36] Tominaga K Shinkai T Eguchi K Saijo N Sasaki Y Beppu Y. The Value of Two-Dimensional Echocardiography in Detecting Malignant Tumors in the Heart. Cancer (1986) 58(8):1641–7. doi: 10.1002/1097-0142(19861015 3463393

[37] Chalmers N Campbell IW. Left Atrial Metastasis Presenting as Recurrent Embolic Strokes. Br Heart J (1987) 58(2):170–2. doi: 10.1136/hrt.58.2.170 PMC1277298 3620256

[38] Limper AH Prakash UB Kokmen E Callahan MJ. Cardiopulmonary Metastatic Lesions of Osteosarcoma and Associated Cerebral Infarction. Mayo Clin Proc (1988) 63(6):592–5. doi: 10.1016/s0025-6196(12)64889-7 3163745

[39] Khoo V Ngan S Guiney M Lim-Joon D. Acute Vascular Embolus Resulting From Metastatic Endocardial Involvement With Synovial Sarcoma: Report of a Case and Review of the Literature. Australas Radiol (1997) 41(1):49–52. doi: 10.1111/j.1440-1673.1997.tb00469.x 9125069

[40] Locci G Pili A Pais M Cirio E Sanna A. Heart Metastases From Gastric Sarcoma. A Case Report. Ital Heart J (2001) 2(7):556–8. 11501966

[41] Cuadrado M García-Camarero T Expósito V Val-Bernal JF Gómez-Román JJ Garijo MF. Cardiac Intracavitary Metastasis of a Malignant Solitary Fibrous Tumor: Case Report and Review of the Literature on Sarcomas With Left Intracavitary Extension. Cardiovasc Pathol (2007) 16(4):241–7. doi: 10.1016/j.carpath.2007.02.006 17637433

[42] Park YM Shin JO Kim M Kang WC Moon J Chung WJ. Cardiac Metastasis of Leiomyosarcoma Complicated With Complete Atrio-Ventricular Block and Ventricular Tachycardia. Korean Circ J (2016) 46(2):260–3. doi: 10.4070/kcj.2016.46.2.260 PMC4805572 27014358

[43] Martinez C Rana JS Solomon MD. Cardiac Metastasis of Nonvisceral Soft-Tissue Leiomyosarcoma. Rev Cardiovasc Med (2017) 18(2):78–81. doi: 10.3909/ricm0859 29038416

[44] Romero J Zapata I Córdoba S Jimeno JM López-Martín JA Tercero JC. *In Vitro* Radiosensitisation by Trabectedin in Human Cancer Cell Lines. Eur J Cancer (2008) 44(12):1726–33. doi: 10.1016/j.ejca.2008.04.013 18501589

[45] D'Incalci M Badri N Galmarini CM Allavena P. Trabectedin, a Drug Acting on Both Cancer Cells and the Tumour Microenvironment. Br J Cancer (2014) 111(4):646–50. doi: 10.1038/bjc.2014.149 PMC4134488 24755886

[46] Barone A Chi DC Theoret MR Chen H He K Kufrin D. FDA Approval Summary: Trabectedin for Unresectable or Metastatic Liposarcoma or Leiomyosarcoma Following an Anthracycline-Containing Regimen. Clin Cancer Res (2017) 23(24):7448–53. doi: 10.1158/1078-0432.CCR-17-0898 28774898

[47] Germano G Frapolli R Belgiovine C Anselmo A Pesce S Liguori M. Role of Macrophage Targeting in the Antitumor Activity of Trabectedin. Cancer Cell (2013) 23(2):249–62. doi: 10.1016/j.ccr.2013.01.008 23410977

[48] Nakamura T Matsumine A Sudo A. The Value of Trabectedin in the Treatment of Soft Tissue Sarcoma. Ther Clin Risk Manage (2016) 12:73–9. doi: 10.2147/TCRM.S84789 PMC4716771 26834480

[49] Gronchi A Hindi N Cruz J Blay JY Lopez-Pousa A Italiano A. Trabectedin and RAdiotherapy in Soft Tissue Sarcoma (TRASTS): Results of a Phase I Study in Myxoid Liposarcoma From Spanish (GEIS), Italian (ISG), French (FSG) Sarcoma Groups. EClinicalMedicine (2019) 9:35–43. doi: 10.1016/j.eclinm.2019.03.007 31143880 PMC6510725

[50] Martin-Broto J Hindi N Lopez-Pousa A Peinado-Serrano J Alvarez R Alvarez-Gonzalez A. Assessment of Safety and Efficacy of Combined Trabectedin and Low-Dose Radiotherapy for Patients With Metastatic Soft-Tissue Sarcomas: A Nonrandomized Phase 1/2 Clinical Trial. JAMA Oncol (2020) 6(4):535–41. doi: 10.1001/jamaoncol.2019.6584 PMC7042948 32077895

